# Modeling the Impact of Fat Flexibility With Dairy Food Servings in the 2015–2020 Dietary Guidelines for Americans Healthy U.S.-Style Eating Pattern

**DOI:** 10.3389/fnut.2020.595880

**Published:** 2020-10-22

**Authors:** Julie M. Hess, Christopher J. Cifelli, Victor L. Fulgoni

**Affiliations:** ^1^National Dairy Council, Rosemont, IL, United States; ^2^Nutrition Impact LLC, Battle Creek, MI, United States

**Keywords:** dairy, dairy products, dietary guidance, dietary patterns, dietary guidelines for Americans

## Abstract

**Background:** The 2015–2020 Dietary Guidelines for Americans (DGA) recommends consuming low-fat or fat-free dairy foods due to concerns about energy and saturated fat intake. It also recommends consuming no more than 10% of daily calories from saturated fat.

**Objective:** The objective was to assess the impact of replacing one serving of fat-free dairy foods in the Healthy U.S.-Style Eating Pattern (HUSEP) from the DGA with one serving of whole- or reduced-fat dairy foods. We hypothesized that this replacement would keep the HUSEP within calorie, saturated fat, and sodium limits.

**Methods:** Utilizing the same modeling procedures as the 2015–2020 DGA, we assessed the nutrient composition of seven alternative models of the 2000-calorie HUSEP. These models replaced all three servings of dairy foods in the HUSEP with an updated fat-free dairy composite (Model 1) or one of three fat-free dairy servings in the HUSEP with: a whole-fat dairy food composite, a reduced-fat/low-fat dairy food composite, whole milk, reduced-fat milk, whole-fat cheese, or reduced-fat cheese (Models 2–7).

**Results:** In all models, the amount of saturated fat did not exceed 10% of total calories, but the amount of energy increased by 45–94 calories. While still lower than current average intake (3,440 mg/d), sodium amounts in four of the seven models exceeded the 2,300 mg/d recommended intake level.

**Conclusions:** Some reduced- and whole-fat dairy foods, especially milk, can fit into calorie-balanced healthy eating patterns that also align with saturated fat recommendations. Allowing some flexibility in fat level of dairy food servings aligns with the recommendations that calories from solid fats and added sugars are best used to increase the palatability of nutrient-dense foods.

## Introduction

Since 1980, the Dietary Guidelines for Americans (DGA) has cautioned against consuming excess saturated fat. Recommendations to “avoid too much fat, saturated fat, and cholesterol” were part of the 1980 DGA and, since 1985, the DGAs have also recommended limiting intake of saturated fats to <10% of calories per day (1–8). In line with these recommendations, since 1985, the DGAs have recommended consuming low-fat or fat-free dairy foods instead of reduced-fat or whole-fat dairy foods to reduce both saturated fat and caloric intake (1–8). In the 2015 DGA, the Healthy U.S.-Style Eating Pattern (HUSEP) and the Healthy Vegetarian Eating Pattern recommend 3 daily servings (cup-equivalents) of low-fat or fat-free dairy foods for all Americans ages 9 and older, 2½ servings for children 4–8 years, and 2 servings for children 2–3 years (1). These recommendations reflect the importance of the overall nutrient composition of dairy foods and their role in in providing important shortfall nutrients to American children and adults (1).

While the DGA recommends low-fat or fat-free dairy foods, a growing body of recent evidence indicates that consuming whole-fat dairy foods does not lead to increased risk of adverse cardiometabolic outcomes, including obesity (9–13), type 2 diabetes (T2D) (14–22), cardiovascular disease (CVD), and stroke (14, 15, 23–45). In some studies, consuming whole-fat dairy foods was linked to a lower risk of adverse cardiometabolic health outcomes (11, 15, 23, 25, 26, 46, 47). For instance, a 2016 intervention trial found that a modified DASH diet with 2–3 daily servings of whole-fat dairy foods instead of low-fat or fat-free dairy foods lowered blood pressure, reduced blood levels of triglycerides, did not increase total cholesterol or low density lipoprotein cholesterol (LDL-C), and also did not decrease high density lipoprotein cholesterol levels (HDL-C), effects similar to those observed with a standard DASH diet containing low-fat or fat-free dairy foods (48).

Further, flexibility in dairy product selection would give Americans more options to meet dairy recommendations and nutrient needs. Accordingly, this study assessed the impact of replacing one of the three servings of low-fat or fat-free dairy foods in the 2000 calorie 2015 DGA's HUSEP with a serving of whole-fat or reduced-fat dairy foods using food pattern modeling. We hypothesized that, using the same food pattern modeling procedures as the 2015 DGA, replacing one serving of the fat-free dairy food composite with one serving of a whole- or reduced-fat dairy food or dairy food composite in the HUSEP would still result in an eating pattern within calorie, saturated fat, and sodium limits.

## Materials and Methods

The 2015 DGA used food pattern modeling to develop recommended healthy eating patterns that could meet Americans' nutrient needs while staying within limits for calories, added sugars, sodium, and saturated fat. These models used a fat-free dairy food composite to estimate the contributions of dairy foods (milk, cheese, and yogurt) to the healthy eating patterns of Americans. The “dairy food composite” is a calculated nutrient profile generated with nutrient data for representative forms of nutrient-dense dairy foods, weighted to match the proportional intake of each food by the U.S. population (49). This dairy food composite reflects actual consumption, based on data from the National Health and Nutrition Examination Survey (NHANES), in terms of the amounts of different dairy foods consumed, but not the actual fat content of the foods consumed. While Americans consume primarily reduced-fat or whole-fat dairy foods (50), the dairy foods used to model eating patterns in the 2015–2020 DGA were almost entirely fat-free products, including fat-free ice cream, cheese, and frozen yogurt (49). Utilizing the same food pattern modeling procedures used for the 2015 DGA, we assessed the energy and nutrient composition of seven alternative models of the 2000-calorie HUSEP, detailed below.

### Dairy Food Composites

To reflect shifts in consumption of different types of dairy foods since the 2010 NHANES, we utilized data from NHANES 2013–2016 to both update a fat-free dairy food composite, similar to the composite used in the 2015 DGA food pattern modeling, and to generate whole- and reduced-fat dairy food composites.

The dairy food composite used in the 2015 DGAC was comprised of 51% milk, 2.6% yogurt, 44.8% cheese, and 1.5% soymilk and utilized primarily fat-free products. The amounts of different products reflects dairy food consumption trends from 2 days of NHANES 2009–2010 consumption data analyzed with USDA Food and Nutrient Database for Dietary Studies (FNDDS), 5.0 (51). Using consumption data from NHANES 2013–2016, the updated fat-free dairy food composite (Composite A) included 47.76% milk, 47.30% cheese, and 4.93% yogurt (Table 1). NHANES 2013–2016 data for Americans ages two and older includes data from *n* = 15,782 Americans, after exclusions for unreliable dietary recalls and data from pregnant and lactating females. Nutrition information for the products used in composites developed for this study are from the updated USDA FNDDS, 2013–2014 and 2015–2016 (52, 53).

**Table 1:** Fat-free, reduced-fat and whole-fat dairy food composites nutrient profile.

	**2015 USDA Dairy Food Composite**	**DAIRY COMPOSITES (47.76% Milk+** **47.30% Cheese** **+** **4.93% Yogurt)^i^**		
		**Composite A**	**Composite B**	**Composite C**
	**(see Table E3.1.A2 from the 2015–2020** **DGA for full list of representative foods used to develop this composite)**	**Fat-free milk (11113000)^ii^ Fat-free cheese (14410130)** **Fat-free yogurt (11411300)**	**Reduced-fat milk (11112110) Reduced-fat cheese (14410120) Low-fat yogurt (11411200)**	**Whole-fat milk (11111000)** **Whole-fat cheese (14410110)** **Whole-fat yogurt (1141100)**
Calories, kcal	77	78	127	157
Protein, g	8.7	10	8.9	8.2
Carbohydrate, g	8.4	9.3	9.0	8.3
Total fat, g	0.9	0.1	6	10
Saturated fats, g	0.6	0.1	3.9	5.7
Cholesterol, mg	7.0	9.3	24	33
**MINERALS**				
Calcium, mg	295	367	295	501
Magnesium, mg	20	45	23	22
Phosphorus, mg	232	215	337	307
Potassium, mg	235	309	272	245
Sodium, mg	202	399	374	387
Zinc, mg	1.1	1.7	1.3	1.1
Selenium, mg	6.6	7.8	6.5	8.7
**VITAMINS**				
Vitamin A, mg_RAE	98	85	130	126
Vitamin E, mg AT	0	0.1	0.1	0.3
Vitamin D, IU	59.3	61.4	116	133
Vitamin C, mg	0.1	0.1	0.3	0.1
Thiamin, mg	0.1	0.2	0.1	0.1
Riboflavin, mg	0.3	0.4	0.4	0.3
Niacin, mg	0.2	1.6	0.2	0.2
Vitamin B-6, mg	0.1	0.2	0.1	0.1
Vitamin B-12, mg	0.8	1.1	1.0	1.0
Choline, mg	24	30	30	27
Vitamin K, mg	0	0.1	0.9	1

i *Composite weighting based on intake percentages normalized to total 100%*.

ii *Numbers in composite denote Food Codes from USDA FoodData Central Database https://fdc.nal.usda.gov/index.html*.

In addition to an updated fat-free composite, two additional dairy food composites were generated for the purposes of this study. Composite B includes reduced-fat milk, reduced-fat cheese, and low-fat yogurt. Low-fat yogurt was used in lieu of reduced-fat yogurt for this composite, because the USDA FNDDS does not contain reduced-fat yogurts. Composite C includes whole-fat milk, whole-fat cheese, and whole-fat yogurt (Table 1), in the proportions consumed by Americans in 2013–2016 NHANES data.

### Modeling

The following models (Tables 2,3) were developed to showcase the impact of one serving of reduced-fat or whole-fat dairy foods in the 2015 DGA's 2000 kcal HUSEP. Model 1 was developed to show the impact of an updated dairy food composite on the HUSEP and to serve as a baseline model for comparison. Models 2–7, also described below, illustrate the impact of replacing a serving of this updated dairy food composite with a serving of a reduced-fat dairy food composite, a whole-fat dairy food composite, whole milk, reduced-fat milk, whole-fat cheese, or reduced-fat cheese. Nutritional information on all dairy foods used for these models comes from FNDDS (53).

**Table 2:** Impact of replacing USDA dairy composite in USDA 2,000 kcal Food Patterns with fat-free, reduced-fat and whole-fat dairy food composites from Table 1 (Models 1–4).

		**MODEL 1**	**MODEL 2**		**MODEL 3**		**MODEL 4**	
	**USDA Food Pattern**	**USDA Food Pattern with 3 servings of Composite A**	**USDA Food Pattern with 2 servings of Composite A and 1 serving of Composite B**	**% change from MODEL 1**	**USDA Food Pattern with 2 servings of Composite A and 1 serving of Composite C**	**% change from MODEL 1**	**USDA Food Pattern with 2 servings of Composite A and 1 serving of whole milk**	**% change from MODEL 1**
**MACRONUTRIENTS**								
Calories, kcal	2,003	2,006	2,055	2.4	2,084	3.9	2,074	3.4
Protein, g	91	95	94	−1.1	93	−1.9	92	−2.6
Carbohydrate, g	256	259	258	−0.1	258	−0.4	261	0.9
Fiber, total dietary, g	31	30	30	0	30	0	30	0
Total lipid (fat), g	72	70	76	8.6	80	14.3	77	11.0
Saturated fats, g	18.7	17.3	21.1	21.9	22.9	32.7	21.7	25.5
Monounsaturated fats, g	26.2	25.5	27.3	6.8	28.0	9.7	27.5	7.5
Polyunsaturated fats, g	22.5	22.3	22.5	0.9	22.8	2.2	22.8	2.2
Linoleic acid (18:2), g	19.6	19.5	19.6	0.7	19.8	1.8	19.8	1.5
Linolenic acid (18:3), g	2.3	2.3	2.3	2.1	2.4	4.9	2.5	7.9
EPA (20:5 n-3), g	0.07	0.07	0.07	0	0.07	3.0	0.07	0
DHA (22:6 n-3), g	0.15	0.15	0.15	0	0.15	0.4	0.15	0
Stearic Acid (18:0), g	4.65	4.39	5.09	16.0	5.41	23.3	5.25	19.7
Cholesterol, mg	215	222	236	6.5	246	10.8	237	6.6
**MINERALS**								
Calcium, mg	1274	1491	1419	−4.8	1624	9.0	1395	−6.4
Iron, mg	17	17	17	0.2	17	1.4	17	0.2
Magnesium, mg	352	426	404	−5.1	403	−5.4	405	−4.8
Phosphorus, mg	1717	1667	1789	7.3	1758	5.5	1653	−0.8
Potassium, mg	3348	3571	3534	−1.0	3507	−1.8	3578	0.2
Sodium, mg	1787	2378	2353	−1.1	2366	−0.5	2082	−12.4
Zinc, mg	14	16	15	−2.5	15	−3.8	15	−4.9
Copper, mg	1.4	1.9	1.7	−7.7	1.8	−6.6	1.8	−5.4
Selenium, mg	110	113	112	−1.1	114	0.8	115	1.0
**VITAMINS**								
Vitamin A, mg_RAE	898	858	903	5.2	899	4.8	885	30
Vitamin E, mg AT	10.2	10.3	10.4	0.3	10.5	2.2	10.4	0.8
Vitamin D, IU	274	280	334	19.3	351	25.4	344	22.6
Vitamin C, mg	117	117	117	0.2	117	0	117	−0.1
Thiamin, mg	1.7	2.0	1.9	−4.9	1.9	−4.9	1.9	−2.7
Riboflavin, mg	2.1	2.4	2.3	−0.7	2.3	−2.3	2.4	1.2
Niacin, mg	24	28	27	−5.0	27	−5.0	27	−4.8
Vitamin B-6, mg	2.3	2.7	2.6	−4.7	2.6	−4.4	2.6	−4.0
Vitamin B-12, mg	6.8	7.7	7.5	−2.1	7.5	−2.2	7.6	−0.6
Choline, mg	349	366	366	0.1	364	−0.6	370	1.3
Vitamin K, mg	139	139	140	0.6	140	0.8	140	0.5

**Table 3:** Impact of replacing USDA dairy composite in USDA 2,000 kcal Food Patterns with fat-free, reduced-fat and whole-fat dairy food composites from Table 1 (Models 5–7).

		**MODEL 1**	**MODEL 5**		**MODEL 6**		**MODEL 7**	
	**USDA Food Pattern**	**USDA Food Pattern with 3 servings of Composite A**	**USDA Food Pattern with 2 servings of Composite A and 1 serving of reduced-fat milk**	**% change from MODEL 1**	**USDA Food Pattern with 2 servings of Composite A and 1 serving of whole-fat cheese**	**% change from MODEL 1**	**USDA Food Pattern with 2 servings of Composite A and 1 serving of reduced-fat cheese**	**% change from MODEL 1**
**MACRONUTRIENTS**								
Calories, kcal	2003	2006	2048	2.1	2097	4.5	2060	27
Protein, g	91	95	93	−2.2	94	−1.2	95	−0.3
Carbohydrate, g	256	259	261	0.9	254	−1.7	255	−1.3
Fiber, total dietary, g	31	30	30	0	30	0	30	0
Total lipid (fat), g	72	70	74	6.7	82	18.1	77	11.0
Saturated fats, g	18.7	17.3	20.2	17.0	24.3	40.7	22.1	27.7
Monounsaturated fats, g	26.2	25.5	26.9	5.1	28.6	12.1	27.8	8.8
Polyunsaturated fats, g	22.5	22.3	22.5	0.8	22.8	2.4	22.5	1.0
Linoleic acid (18:2), g	19.6	19.5	19.6	0.8	19.9	2.2	19.6	0.8
Linolenic acid (18:3), g	2.3	2.3	2.3	0.8	2.3	2.2	2.4	3.6
EPA (20:5 n-3), g	0.07	0.07	0.07	0	0.07	6.3	0.07	0
DHA (22:6 n-3), g	0.15	0.15	0.15	0	0.15	0.7	0.15	0
Stearic Acid (18:0), g	4.65	4.39	4.96	13.0	5.60	27.7	5.26	19.9
Cholesterol, mg	215	222	232	4.5	255	15.2	242	9.0
**MINERALS**								
Calcium, mg	1274	1491	1411	−5.3	1880	26.1	1414	−5.1
Iron, mg	17	17	17	0	17	2.8	17	0.4
Magnesium, mg	352	426	408	−4.3	400	−6.1	399	−6.2
Phosphorus, mg	1717	1667	1673	0.3	1874	12.4	1908	14.4
Potassium, mg	3348	3571	3598	0.8	3424	−4.1	3443	−3.6
Sodium, mg	1787	2378	2092	−12.0	2683	12.8	2640	11.0
Zinc, mg	14	16	15	−3.2	15	−2.8	15	−2.3
Copper, mg	1.4	1.9	1.7	−7.8	1.7	−7.8	1.7	−7.7
Selenium, mg	110	113	112	1.5	115	1.0	113	−0.8
**VITAMINS**								
Vitamin A, mg_RAE	898	858	905	5.5	922	7.4	911	6.2
Vitamin E, mg AT	10.2	10.3	10.3	−0.1	10.7	3.7	10.4	0.7
Vitamin D, IU	274	280	334	19.2	362	29.1	335	19.7
Vitamin C, mg	117	117	117	0.3	117	−0.1	117	−0.1
Thiamin, mg	1.7	2.0	1.9	−3.6	1.8	−7.2	1.9	−6.3
Riboflavin, mg	2.1	2.4	2.4	2.9	2.2	−6.0	2.2	−4.8
Niacin, mg	24	28	27	−4.8	27	−5.2	27	−5.2
Vitamin B-6, mg	2.3	2.7	2.6	−3.9	2.6	−4.7	2.6	−5.6
Vitamin B-12, mg	6.8	7.7	7.8	2.0	7.4	−3.7	7.2	−6.7
Choline, mg	349	366	375	2.7	356	−2.7	356	−2.6
Vitamin K, mg	139	139	140	0.3	141	1.2	141	1.0

To further illustrate the differences between the Model 1 baseline and Models 2–7, percent change was calculated for each nutrient. Nutrition labeling in the U.S. indicates that foods providing between 10 and 19% of the Daily Value (labeling value derived from recommended reference intakes) of a given nutrient can claim to be a “good source” of that nutrient. Foods providing 20% or more of the Daily Value of a given nutrient can claim they are an “excellent source” (54). Therefore, in this study, 10 and 20% differences indicate meaningful changes from the Model 1 baseline (55, 56).

Model 1: Replace all 3 cup equivalents of the USDA's dairy food composite with 3 servings of Composite A.
Model 2: Replace 2 cup equivalents of the USDA's dairy food composite with 2 servings of Composite A and 1 serving of Composite B.
Model 3: Replace 2 cup equivalents of the USDA's dairy food composite with 2 servings of Composite A and 1 serving of Composite C.
Model 4: Replace 2 cup equivalents of the USDA's dairy food composite with 2 servings of Composite A and 1 serving of whole milk (11111000).
Model 5: Replace 2 cup equivalents of the USDA's dairy food composite with 2 servings of Composite A and 1 serving of reduced-fat milk (11112110).
Model 6: Replace 2 cup equivalents of the USDA's dairy food composite with 2 servings of Composite A and 1 serving of whole-fat cheese (14410110).
Model 7: Replace 2 cup equivalents of the USDA's dairy food composite with 2 servings of Composite A and 1 serving of reduced-fat cheese (14410120).

## Results

Tables 2–4 show the impact of these models on macronutrient and micronutrient amounts and their comparison with dietary goals (i.e., % Recommended Dietary Allowance). For this study, sample age/sex groups were females 19–30 years of age and males 51+ years of age. Tables 2,3 also show percent change between Model 1 and Models 2–7.

**Table 4:** Comparing food pattern modeling from Tables 2, 3 to dietary goals for sample age/sex groups with 2,000 kcal diets, taken from Table E3.1.A4 of 2015 DGAC Scientific Report (% goal; % Recommended Dietary Allowance, RDA; % Adequate Intake, AI; % Upper Limit, UL; %kcal).

	**MODEL 1^iii^**		**MODEL 2^iv^**		**MODEL 3^v^**		**MODEL 4^vi^**		**MODEL 5^vii^**		**MODEL 6^viii^**		**MODEL 7^ix^**	
**Nutrients, % goal**	**Females 19–30 years**	**Males 51+ years**	**Females 19-30 years**	**Males 51+ years**	**Females 19-30 years**	**Males 51+ years**	**Females 19-30 years**	**Males 51+ years**	**Females 19-30 years**	**Males 51+ years**	**Females 19-30 years**	**Males 51+ years**	**Females 19-30 years**	**Males 51+ years**
**MACRONUTRIENTS**														
Kcal, % goal	100	100	103	103	104	104	104	104	102	102	105	105	103	103
Protein, % RDA	207	170	204	167	202	166	201	165	202	166	204	167	205	169
Protein, % kcal	19	19	18	18	18	18	18	18	18	18	18	18	18	18
Carbohydrate, % RDA	199	199	199	199	198	198	201	201	201	201	196	196	196	196
Carbohydrate % kcal	52	52	50	50	49	49	50	50	51	51	48	48	50	50
Fiber, % AI	109	109	109	109	109	109	109	109	109	109	109	109	109	109
Saturated fat, % kcal	8	8	9	9	10	10	9	9	9	9	10	10	10	10
Monounsaturated fat, % kcal	11	11	12	12	12	12	12	12	12	12	12	12	12	12
Polyunsaturated fat, %vkcal	10	10	10	10	10	10	10	10	10	10	10	10	10	10
Linoleic acid, % AI	162	139	164	140	165	142	165	141	164	140	166	142	164	140
Linolenic acid, % AI	207	142	211	145	217	149	223	153	208	143	211	145	214	147
**MINERALS**														
Calcium, % RDA	149	124	142	118	162	135	139	116	141	118	188	157	141	118
Iron, % RDA	94	210	94	211	95	213	94	211	94	210	96	216	94	211
Magnesium, % RDA	137	101	130	96	130	96	131	96	132	97	129	95	129	95
Phosphorus, % RDA	238	238	256	256	251	251	236	236	239	239	268	268	273	273
Potassium, % AI	76	76	75	75	75	75	76	76	77	77	73	73	73	73
Sodium, % UL	103	103	102	102	103	103	91	91	91	91	117	117	115	115
Zinc, % RDA	196	142	191	139	189	137	186	135	190	138	190	138	191	139
Copper, % RDA	210	210	193	193	196	196	198	198	193	193	193	193	193	193
Selenium, % RDA	206	206	204	204	208	208	208	208	203	203	208	208	205	205
**VITAMINS**														
Vitamin A, % RDA	123	95	129	100	128	100	126	98	129	101	132	102	130	101
Vitamin E, % RDA	69	69	69	69	70	70	69	69	69	69	71	71	69	69
Vitamin D, % RDA	47	47	56	56	59	59	57	57	56	56	60	60	56	56
Vitamin C, % RDA	156	130	156	130	156	130	156	130	157	130	156	130	156	130
Thiamin, % RDA	180	165	171	157	171	157	175	161	174	159	167	153	169	155
Riboflavin, % RDA	214	181	213	180	209	177	217	184	221	187	201	170	204	173
Niacin, % RDA	202	177	192	168	192	168	192	168	192	168	191	167	191	167
Vitamin B6, % RDA	208	159	199	152	199	152	200	153	200	153	198	152	197	150
Vitamin B12, % RDA	320	320	313	313	313	313	318	318	326	326	308	308	299	299
Choline, % RDA	86	66	86	67	86	66	87	67	88	68	84	65	84	65
Vitamin K, % RDA	155	116	156	117	156	117	155	117	155	116	157	117	156	117

iii *USDA Food Pattern with 3 servings of Composite A replacing 3 servings of 2015-2020 Dietary Guidelines for Americans dairy food composite*.

iv *USDA Food Pattern with 2 servings of Composite A and 1 serving of Composite B replacing 3 servings of 2015-2020 Dietary Guidelines for Americans dairy food composite*.

v *USDA Food Pattern with 2 servings of Composite A and 1 serving of Composite C replacing 3 servings of 2015-2020 Dietary Guidelines for Americans dairy food composite*.

vi *USDA Food Pattern with 2 servings of Composite A and 1 serving of whole milk (**11111000**) replacing 3 servings of 2015-2020 Dietary Guidelines for Americans dairy food composite*.

vii *USDA Food Pattern with 2 servings of Composite A and 1 serving of reduced-fat milk **(11112110)** replacing 3 servings of 2015-2020 Dietary Guidelines for Americans dairy food composite*.

viii *USDA Food Pattern with 2 servings of Composite A and 1 serving of whole-fat cheese **(14410110)** replacing 3 servings of 2015-2020 Dietary Guidelines for Americans dairy food composite*.

ix *USDA Food Pattern with 2 servings of Composite A and 1 serving of reduced-fat cheese (**14410120)** replacing 3 servings of 2015-2020 Dietary Guidelines for Americans dairy food composite*.

The amount of energy increased across all models, since no other modifications to the eating pattern were made (Tables 2,3), though none of the increases exceeded 5%. The percent change in saturated fat from Model 1 ranged from an increase of 17.0% in Model 5 to an increase of 40.7% in Model 6. The percentage of calories from saturated fat increased from 8% of total calories in the original 2000 kcal HUSEP diet and Model 1 but did not exceed 10% of total calories in Models 2–7. In Models 2, 4, and 5, which added one serving of the reduced-fat composite or milk, saturated fat was 9% of total calories. The amount of sodium increased across all models, including Model 1, due to changes in dairy product consumption. While the original 2,000 kcal HUSEP had 1,787 mg of sodium, Model 1 (with 3 servings of updated fat-free Composite A) contained 2,378 mg of sodium. The amount of sodium in some other models remained below the 2,300 mg/d threshold for reducing risk of chronic disease set by the National Academies of Sciences, Engineering, and Medicine in 2019 (57). Models 4 and 5, which replaced a serving of the fat-free dairy food composite with whole- and reduced-fat milk, respectively, had 2,082 and 2,092 mg of sodium, decreases of 12.4 and 12.0%, respectively, from Model 1.

Model 1, which updated the fat-free dairy foods composite used in the original 2,000 kcal HUSEP, led to an eating pattern with 2,006 kcal (compared to 2003 in original HUSEP), 4 more grams of protein, less total fat, saturated fat, and monounsaturated fat, and a 591 mg increase in sodium content. Including one serving of a “reduced-fat dairy foods composite” and 2 servings of an updated fat-free dairy food composite in the 2,000 kcal HUSEP kept saturated fat intake at 9% of total calories (Table 4) and increased energy by 49 kcal (total calories = 2,055 kcal), compared to Model 1 (Table 2). Including one serving of a “whole-fat dairy foods composite” and 2 servings of a “fat-free dairy food composite” as part of the 2000 kcal HUSEP results in saturated fat intake at 10% of total calories (Table 4) and increases total caloric intake by 78 kcal (total calories = 2,084 kcal; ~4% increase), compared to Model 1 (Tables 2,4).

Replacing one serving of the updated dairy food composite with one serving of whole milk or reduced-fat milk in the 2,000 kcal HUSEP increased calories by 68 kcal (3.4% increase) and 42 kcal (2.1% increase), respectively, compared to Model 1. Both models provided 9% of calories from saturated fat (Table 4). Replacing one serving of the updated dairy food composite with one serving of whole-fat cheese or reduced-fat cheese in the 2,000 kcal HUSEP increased calories by 91 kcal (4.5% increase) and 54 kcal (2.7% increase), respectively, compared to than Model 1. Both models had 10% of calories from saturated fat (Table 4).

Vitamin D was higher by ~19–29% in Models 2–7, compared to Model 1. Model 6 had the largest increase in Vitamin D content from Model 1 (29.1%). Model 6 also had >10% increases in calcium and phosphorus compared to Model 1. Model 7 had a 14.4% increase in phosphorus compared to Model 1, but none of the other models had changes that exceeded 10% with either calcium or phosphorus. Changes in the amounts of other minerals (iron, magnesium, potassium, zinc, copper, selenium) and vitamins (vitamins A, E, C, B6, B12, K, thiamin, riboflavin, niacin, and choline) in Models 2–7 did not differ by more than 10% from Model 1.

## Discussion

Food pattern modeling indicates that one of the three recommended servings of dairy foods for Americans 9 years and older can be a whole- or reduced-fat option while staying within the 2015 DGA's recommended ranges for saturated fat, energy, and sodium intake, especially if the choices are reduced-fat or whole-fat milk. The amount of saturated fat in all seven models did not exceed 10% of total calories, the upper limit recommended in the 2015 DGA (1). While shifting from low-fat or fat-free dairy foods to reduced-fat and whole-fat foods increased the energy content of the HUSEP, this increase in calories could be mitigated by decreasing calories from added sugars or refined grains, a dietary strategy that does not significantly impact overall dietary nutrient content and has been used successfully to manage energy intake in intervention trials using whole-fat dairy foods (48). Total calorie allowance for added sugars in a 2,000 kcal eating pattern is 120 kcal (93), and the largest increase in calories in the modeled eating patterns was 91 kcals (Model 6 compared to Model 1). In addition, while the amount of sodium in Models 1, 2, 5, and 6 exceeds the 2,300 mg/d Chronic Disease Risk Reduction Intake level for sodium, the sodium in these models is still lower than the current average sodium intake (3,410 mg/d) of Americans by at least 700 mg (58) (Model 6).

While dairy foods are an important source of nutrients in the diets of American children and adults, including for three of the four nutrients of public health concern (calcium, potassium, and vitamin D) (59–61), most Americans do not meet dairy food recommendations. On average, Americans consume <2 cup equivalents of dairy foods daily (50). In addition to helping Americans meet nutrient needs, meeting recommendations for dairy foods may also lead to healthcare cost savings up to $12.5 billion for the U.S., due to projected reductions in stroke, hypertension, type 2 diabetes, and colorectal cancer from adequate intake (62). While more targeted research is needed, flexibility with the fat level of dairy food servings in dietary guidance could help Americans meet recommendations, since most of the dairy foods that Americans currently consume are reduced-fat or whole-fat products (50). Allowing flexibility in dairy food servings also aligns with the recommendation from the 2010 DGA that calories from solid fats and added sugars are best used to increase the palatability of nutrient-dense foods, which includes milk, cheese, and yogurt (8).

Flexibility with whole- and reduced-fat dairy foods in the DGA would accurately reflect the latest scientific evidence on whole-fat and reduced-fat dairy foods and cardiometabolic outcomes. Two other authoritative health organizations have recently published dietary recommendations with “fat flexibility” with dairy food selection within healthy eating patterns. In 2016, Joslin Diabetes Center's Nutrition Guidelines for Overweight and Obese Adults with T2D or Prediabetes, or Those at High Risk for Developing T2D states “recent evidence demonstrates that saturated fat from dairy foods (milk, yogurt, cheese) may be acceptable within the total daily caloric intake” (63). Similarly, in 2019, the Australian Heart Foundation's (AHF) statement on heart-healthy eating patterns encouraged consumption of dairy foods, regardless of fat content, as part of healthy eating patterns. The AHF stated that “there is not enough evidence to recommend fat modification (full-fat over reduced-fat products or reduced-fat over full-fat products) for the general population” (64).

One reason that whole- and reduced-fat dairy foods may have different effects on LDL-C than would be expected based on their saturated fat content is due to the complexity of dairy fat (65). Approximately two-thirds of milkfat is saturated fatty acids (including short-, medium-, long-, branched-, and odd-chain fatty acids) and one-third is unsaturated fat (including mono- and polyunsaturated fatty acids). Dairy fat contains over 400 types of fatty acids that have different physiological effects (65). Stearic acid, which contains 18 carbons, has no effect on LDL-C (66). The other most common saturated fatty acids in dairy fat (12, 14, or 16 carbons) raise blood levels of LDL-C but also raise HDL-C levels and lower triglyceride levels, a pattern associated with lower risk for CVD (66, 67). Dairy fat also contains small amounts of saturated fatty acids with 15 and 17 carbons. These fatty acids have also been associated with reduced risk of CVD and T2D (17, 46).

Food pattern modeling indicates that the HUSEP from the 2015 DGA with one serving of whole- or reduced-fat dairy foods and two servings of low-fat or fat-free dairy foods can be accomplished within calorie and nutrient recommendations. The methods in this study are subject to limitations, however. Because this study relied on food pattern modeling, it is theoretical in nature and not developed from actual eating patterns. The modeling results indicate that, of the three servings per day of recommended for Americans 9 years and older, one serving of whole- or reduced-fat dairy foods, such as a glass of whole milk or serving of reduced-fat cheese, can be part of healthy eating patterns for Americans.

## Data Availability Statement

All datasets used in this study are publicly available and references in the article indicate how to access them.

## Author Contributions

CC and VF: conceptualization and writing- review and editing. JH, CC, and VF: methodology. JH: writing- original draft preparation. Final manuscript was read and approved by all authors.

## Conflict of Interest

JH and CC work for National Dairy Council. VF III, as senior vice-president of Nutrition Impact LLC, performs consulting and database analyses for various food and beverage companies and related entities including the National Dairy Council.
